# Paving the Way to Eureka—Introducing “Dira” as an Experimental Paradigm to Observe the Process of Creative Problem Solving

**DOI:** 10.3389/fpsyg.2018.01773

**Published:** 2018-10-02

**Authors:** Frank Loesche, Jeremy Goslin, Guido Bugmann

**Affiliations:** ^1^CogNovo, Cognition Institute, Plymouth University, Plymouth, United Kingdom; ^2^School of Computing, Electronics and Mathematics, Plymouth University, Plymouth, United Kingdom; ^3^School of Psychology, Plymouth University, Plymouth, United Kingdom

**Keywords:** creative problem solving, divergent thinking, convergent thinking, behavioral experimental paradigm, chronometric temporal measures, insight, chronology

## Abstract

“Dira” is a novel experimental paradigm to record combinations of behavioral and metacognitive measures for the creative process. This task allows assessing chronological and chronometric aspects of the creative process directly and without a detour through creative products or proxy phenomena. In a study with 124 participants we show that (a) people spend more time attending to selected vs. rejected potential solutions, (b) there is a clear connection between behavioral patterns and self-reported measures, (c) the reported intensity of Eureka experiences is a function of interaction time with potential solutions, and (d) experiences of emerging solutions can happen immediately after engaging with a problem, before participants explore all potential solutions. The conducted study exemplifies how “Dira” can be used as an instrument to narrow down the moment when solutions emerge. We conclude that the “Dira” experiment is paving the way to study the process, as opposed to the product, of creative problem solving.

## 1. Introduction

Creativity (Runco and Acar, 2012), innovation (Amabile, 1988), and problem solving (Newell and Simon, 1972) have shaped human history, culture, and technology. Valued by today's society for their contributions to education, recruiting, and employment (Cropley, 2016) they are also likely to play an essential role in our future society. Moreover, creativity, innovation, and problem solving are required to address the increasingly complex problems we are facing. A commonality between these phenomena is the aim of identifying novel and useful answers to more or less well-defined and ill-defined questions (Simon, 1973; Weisberg, 2006). Based on observations and reports from eminent scientists such as Helmholtz and Poincaré, Wallas (1926) famously suggested that the process of generating answers or creative products consists of several consecutive phases. Since then the exact structure and number of these stages are being debated (Amabile, 1983; Finke, 1996; Csikszentmihalyi, 2009; Amabile and Pratt, 2016), but arguably, the moment when a solution emerges lies at the heart of the matter. This “illumination” phase often follows and precedes other stages (Howard et al., 2008): Before finding the solution, the problem solver needs to “prepare” for the problem at hand, for example by understanding the question, potentially within the larger context. If people do not solve the problem in this phase, they might enter a stage of “incubation.” In this stage, they are thought to unconsciously keep processing the problem while they consciously attend to other tasks. The feeling of manifesting associations or fringe consciousness coined as “intimation” is the next stage in this model (Sadler-Smith, 2015). Following this, the problem solvers experience a phase of “illumination” when they suddenly have an idea that answers the question. Afterwards, during the “verification” stage, this solution is tested. Certain models consider additional stages to communicate and implement a found solution as part of the process. Csikszentmihalyi (2009), for example, calls it the “elaboration” stage. To sum up, within existing case studies of creativity, innovation, and problem solving and the theories behind them, the moment when solutions emerge is part of a longer “creative process.” However, most studies focused on the outcome of these three phenomena, without considering the various processes behind them.

Previous studies identify the moment when solutions emerge through a range of different phenomena (Kounios and Beeman, 2014), for example restructuring the problem representation (Knoblich et al., 1999; Fleck and Weisberg, 2004), an alteration of mood (Baas et al., 2008; Subramaniam et al., 2009), and the suddenness of changes (Topolinski and Reber, 2010a). Reports of these potentially associated phenomena have been used as markers of “insights,” “Aha! moments,” and “Eureka experiences.” However, some of these phenomena might only be weak proxies. Danek et al. (2016) have shown that not every solved problem relies on restructuring. In a follow-up study, Danek and Wiley (2017) revealed that not every experience of insight results in a solved problem. Even if a link between observed phenomenon and “Eureka experience” is well established as for the mood change, the chronology or even causality remains unclear: Does insight increase mood (Akbari Chermahini and Hommel, 2012), does a stimulated positive mood cause “Aha! moments” (Isen et al., 1987; Ritter and Ferguson, 2017), or are they both results of another process? Therefore, there is a need to detect emerging solutions directly and not via proxy phenomena. Moreover, most studies on insight assume Eureka experiences are dichotomous, “Aha! moments” either suddenly happen or not (Bowden and Jung-Beeman, 2003; Gilhooly and Murphy, 2005; Subramaniam et al., 2009; Hedne et al., 2016). Possibly the phenomenon benefits from a more differential view, theoretically and empirically.

**Figure 1:**
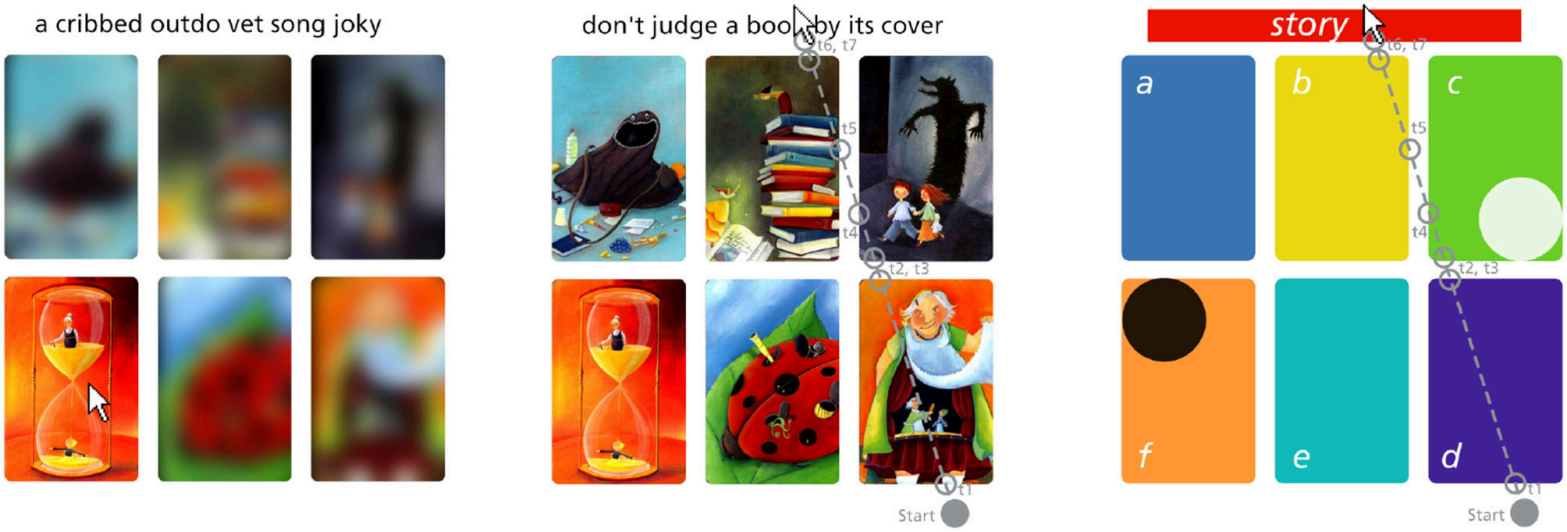
Example of the screen during a “quiz” **(Left)**, all elements unblurred **(Center)**, and the color coded positions **(Right)**. The center and right subfigure show an example mouse movement. The mouse positions at onset and offset times *t*_1_ to *t*_7_ are recorded as raw data. The figure on the right shows assigned symbolic names and colors for each position “a”–“f” and “story” (text) as used in later plots. The text was initially inspired by the image with the white circle, because in “image c” the shadow reveals the true intention of the figures in the foreground. The black circle marks an example of a “chosen solution”. Dixit images by Libellud.

In this paper, we introduce “Dira” as a novel experimental paradigm to narrow down the moments of emerging solutions within the creative process. In each of the forty “Dira” tasks, participants are asked to find a solution. A solution is the image they consider to correspond best with a one-line text. On a computer display, the on-screen text and images appear blurred by default and can only be seen clearly when the mouse hovers above them (see Figure 1). Tracing the mouse movement and the hover time on each image allows to measure the time participants spend processing an image during task execution and before they report a solution. After each task participants provide metacognitive self-reports, such as the intensity of their Eureka experience that accompanies emerging solutions (Cushen and Wiley, 2012; Danek et al., 2014). We hypothesize that the combination of behavioral measures of the process and self-reports can be used to identify distinctive behaviors when solutions emerge and localize the solutions' emergence in time. Further, we hypothesize that feedback on the participants' choice moderates the behavior and the reported Eureka experience thereafter.

## 2. Rationale

In this section, we summarize existing tasks that have been used to observe the moment solutions emerge during creative problem solving and we provide an argument for a novel experimental paradigm. We describe the origin of “Dira” and how we acquired the problems participants are asked to solve. Finally, we argue for the mouse-tracking method to trace people's problem solving process.

### 2.1. Existing tasks related to emerging solutions

Different types of tasks have traditionally been associated with the creative process and emerging solutions, namely insight tasks, divergent thinking tasks, and convergent thinking tasks.

From a historical perspective, insight tasks (Maier, 1930; Duncker, 1963; Gardner, 1978; MacGregor et al., 2001) are the oldest of these types of tasks. They predate the distinction between divergent and convergent production as introduced by Guilford (1967) and were consequently developed without a direct reference to one of these processes. These insight tasks often take the form of riddles or visual puzzles and are built around the assumption that the task itself requires restructuring (Knoblich et al., 1999; Fleck and Weisberg, 2004). The overlap between insight tasks and convergent thinking tasks seem particularly strong: for example, Bowden and Jung-Beeman (2003) argue, that convergent thinking problems like the Remote Associate Task share properties with insight tasks. Nevertheless, convergent thinking tasks can either be solved via insight or without. Similarly, classical insight problems are often thought to converge to a single solution, even though examples for the nine-dot problem show that more than one solution is possible (Maier, 1930; Sarcone, 2014). Furthermore, and as Bowden et al. (2005) and Danek et al. (2016) demonstrate, finding solutions to insight tasks does not require insight or an Aha experience. While timing has been discussed since the earliest studies on insight tasks, often it only relates to the time when a solution is found. These type of tasks are not repeatable and allow only between-subject comparisons. Even more, having solved similar problems in the past seems to influence the process (Lung and Dominowski, 1985), and it is difficult to identify the similarity between problems as well as to control for previous exposure. Consequently, the classic insight problems are not considered for this study.

Divergent thinking tasks (Torrance, 1966; Guilford, 1967; Runco et al., 2016), in which people are asked to generate several potential solutions to a question, are associated with individual creative processes. Nevertheless, the measurement of originality is usually assessed within the cohort of the experiment and not for an isolated individual. Consider a “Brick Uses” task (Wilson et al., 1954; Guilford, 1967, p. 143) in which participants are asked for alternative uses of a brick. An answer to use the brick's pigments to paint might be unique within an experiment, but the participant might just have reported an instance from memory (Gilhooly et al., 2007; Hass, 2017). Hence this solution, although original within the experiment, did not require creative problem solving from this particular individual. Furthermore, before assessing the originality, raters decide if answers are considered for the scoring. For the answer “to paint” in a “Brick Uses” task, which is similar to the previous example, some would consider it an “impossible answer” and consequently remove the answer before scoring originality. Time measurements are often provided by a minimum or maximum task time and through fluency measures, and recently the moments of the production of a solution have received more attention (Forthmann et al., 2017). Divergent thinking tasks are in general repeatable, but the difficulty in scoring, and the unknown origin of the solution, either from memory or as a novel product, disqualify these types of tasks for our purpose.

Finally, Convergent thinking tasks (Mednick, 1962; Knoblich et al., 1999; Bowden and Jung-Beeman, 2003), require participants to come up with a single solution. These tasks are based on the difficulty to search a large problem space, produce interim solutions, and verify these results. Some of these tasks, such as the Compound Remote Associates test, were developed to specifically address the shortcomings of the classical insight tasks (Bowden and Jung-Beeman, 2003). Convergent thinking tasks typically provide a large number of stimuli for repeated measures. For word-based convergent thinking problems, language fluency affects the ability to solve the problem (Hommel et al., 2011).

In our study, we intended to observe behavior during the creative process, but for problems with three verbal stimuli such as the Compound Remote Associate task, prospective problem solvers might not exhibit much observable behavior. The low number of word-based stimuli within a single task (typically three) are easy to memorize, and participants can operate entirely on their working memory. There is little incentive to reread the words or exhibit other behavioral cues through which the internal thought process could be traced. The timing of the solution and the success within a given time are central measurements in this type of task. For example, Salvi et al. (2016) ask their participants to press a button as soon as they found a solution. This timing relates only to the whole process but does not allow the identification of the involved sub-stages. Therefore we decided not to use convergent thinking tasks to trace the emerging solution within the creative problem solving process.

### 2.2. Development of “dira”

“Dira” has been developed out of the necessity to collect fine-grained measurements of the creative process. As an experimental paradigm to observe the moment when solutions emerge, “Dira” needs to address one fundamental requirement: the solution should not be known from the beginning. In this sense, a solution could either be the answer itself or an algorithm how to arrive at the answer. If either was known at the moment the task was given, “Dira” would merely provide measures related to other processes, for example processing fluency and memory retrieval.

“Dira” is inspired by “Dixit,” a commercially available and internationally acclaimed card game. The word “Dixit” is Latin for “he or she said,” chosen by the French developers of the game, supposedly to highlight the story-telling aspect. We use the French word “Dira” for “he or she will point out” as a reference to the process throughout the task as well as the origin of the inspiring game. The 84 unique images of a “Dixit” card deck are described as “artwork”^1^ and “dreamlike”^2^ and have previously been used in teaching a foreign language (Cimermanová, 2014), in research on imaginative design narratives (Berger and Pain, 2017), and observing conformity and trust between humans and robots (Salomons et al., 2018). The cards have also inspired interventions to foster creativity (Liapis et al., 2015), and are suggested as “an additional source of inspiration” (Wetzel et al., 2017, p. 206) for an ideation method.

The task “Dira” we developed uses elements and data from the game “Dixit.” Therefore, we briefly introduce some relevant aspects of the game. Three to six players can participate in the “Dixit” game, which is played in several rounds. At the beginning of a round, one of the players is appointed as the storyteller. From the deck of 84 unique cards with beautifully drawn images, each player receives six cards in their hand. Based on the drawing on one of the cards, the storyteller invents a short text and tells it to the other players. Related to this text, all other players select one card from their hand. The selected cards are shuffled and played on the table. Now all players except the storyteller have to guess which of the images originally inspired the text. Based on their choice, the storyteller and all other players receive points. Hereby the scoring system penalizes storyteller that produce descriptive texts and associations that are easy to find. Furthermore it encourages the others to play cards with a similar non-obvious connection to the text. Moreover, and based on the different associations the players formed, each image has some connection to the text. At the end of a round, a group of players has produced a combination of a short text and as many associated images as there are players. Nevertheless, and as the example in Figure 1 illustrates, it would defy the purpose of the game if the other players would immediately understand any of these connections.

In each “Dira” task we ask people to find a connection between a short text and one of six images sampled from past “Dixit” games with six players. As argued before, people are unlikely to identify the image that inspired the text immediately. Instead, they might find a connection between the text and one of the six potential solutions through controlled processes in creative cognition (Beaty and Silvia, 2012; Silvia et al., 2013) or unconscious associations (Mednick, 1962; Kenett et al., 2014). In the first case, participants generate several metaphors or potential solutions from available information and select one of them as the best fit at a specific time. In the second case, existing associations are mediated through similarities of common elements before one of them is identified as the best match. In both cases, the solution emerges at a distinct moment before participants select one image by a mouse click. Participants in the “Dira” task are forced to make a choice, but which of the six possible solutions they choose depends on their prior knowledge and their subjective understanding of the task at hand. These differences in problem difficulty are described for other problems as well. Often, the correctness of a task solution is considered vital to the measures and consequently needs to be controlled for, as Öllinger et al. (2014) demonstrate for a well know 9-dot problem. “Dira” does not have one objectively correct solution and we are not interested in the exact timing of finding the subjectively correct solution. Instead, we assess the behavior during the process through the interaction times with text and images.

For the developed task we assume that two different modalities for the stimuli are advantageous to isolate remote conceptual associations. If the two stimuli that were to be matched used the same modality, matches could be found for aspects of these stimuli that are outside the interest of this study. For example matches between two visual stimuli could not only be based on the depicted content, but also on colors, forms, and dynamics of the image. For two verbal stimuli the constructing syllables, cultural connotations, and language fluency of the problem solver would play a decisive role in the selection of an answer. By asking people to match content from different modalities, we hope to circumvent the issues above.

### 2.3. Dataset

The experience of an emerging solution relies on the inherent quality of the task; in the case of “Dira” on the text as well as on each of the potentially associated images. Instead of constructing a synthetic dataset, we crowdsourced the combination of a single text and six accompanying images from a community of experienced “Dixit” players. Usually, the card game “Dixit” is played locally around a table. For groups not sharing the same space, Boite-a-jeux^3^ provides an online gaming platform to play this game across distances and with other players of a similar skill level. In August 2014 we accessed the publicly available recorded game data of 115,213 rounds of “Dixit.” We filtered this initial dataset for English rounds with six players. After stopword removal (such as “the,” “is,” “at”) and word stemming, we removed the rounds with stories containing the most frequent words from the 90th percentile. Looking at the text and images, candidate sets for the “Dira” task were selected from the remaining 1,000 rounds of recorded “Dixit” games. The authors of this paper, two of which are experienced “Dixit” players, chose 40 combinations of text and images. Afterwards, we identified between one and three contexts of associated knowledge to control for participants' domain-specific knowledge in a later analysis. For example, the sentence “Standing on the shoulders of giants” is meaningful in different domains like the scientific community exposed to life and work of Newton, but also for fans of the Britpop group “Oasis,” who released an album with the same name. The identified contexts were then grouped into the following eight clusters (with the number of associated stories in brackets): Literature (8), music (6), film (7), science (7), popular culture (12), and high culture (7) as well as word games (11), and literal interpretations of visual cues (10). These contexts allow to control for required knowledge to solve the tasks. Finally, the order of the tasks within the “Dira” experiment was initially chosen at random but kept the same throughout all conditions reported in this paper.

### 2.4. Mouse-tracking as process-tracing

“Dira” is based on the fundamental assumption that psychological processes can be traced through observable behavior (Skinner, 1984). Of particular interest to the emerging solutions is the participants' behavior during the task when they are engaged in a creative problem solving process. At the beginning of each task, participants do not know the text or the images. To solve the problem, they have to acquire information from these elements and find associations between the text and the images. For “Dira” the process of information acquisition is related to the order and timing of interactions with each of the elements on the “quiz” screen. Different methods are commonly used to trace these chronology and chronometric measures of processes, for example through verbal protocols (Newell and Simon, 1972), eye-tracking (Thomas and Lleras, 2007), and mouse-tracking (Freeman and Ambady, 2010).

Verbal and think-aloud protocols have been used in insight tasks (Fleck and Weisberg, 2004), divergent thinking tasks (Gilhooly et al., 2007), convergent thinking tasks (Cranford and Moss, 2012), and also in real-world problem solving (Newell and Simon, 1972; Kozbelt et al., 2015). While Schooler et al. (1993) identified an overshadowing effect for insight problem solving, Gilhooly et al. (2007) did not find any effect on fluency and novelty production in a divergent thinking task. In a meta-study, Fox et al. (2011) did not see an effect of verbalization on the results of tasks, but they noted an increase in the time required. These results suggest that think-aloud protocols might or might not change the solutions provided for a task, but they most certainly change the process. With our interest in narrowing down the time of emerging solutions within a process, verbal protocols seemed too invasive and were disregarded.

In a direct comparison between eye-tracking and mouse-tracking, Lohse and Johnson (1996, p. 37) conclude that mouse interactions “predispose people to use a more systematic search and process more information than they normally would.” Similar to the technique described by Ullrich et al. (2003), elements in the “quiz” of “Dira” that are not directly under the mouse pointer are blurred. These indistinct images prevent participants from accessing this information without moving the mouse pointer to an element. A notable difference to the method developed by Ullrich et al. (2003) is that elements in “Dira” do not fade over time; elements are visible for the whole time the mouse pointer hovers over them. Uncovered images imply that information acquisition and information processing is possible throughout the whole hover time. Indeed, participants will not necessarily direct their full attention to the currently unblurred text or image. While this appears as a disadvantage of mouse-tracking, Ferreira et al. (2008) have observed the same issue for eye-tracking. People are also known to not always perceive visual input when generating ideas (Walcher et al., 2017). Furthermore, other processes such as memory access are related to eye movements as well (Johansson and Johansson, 2013; Scholz et al., 2015). Nevertheless, Freeman and Ambady (2010) have shown that mouse-tracking provides reliable insight into mental processes and while it provides more robust measures than eye-tracking, it is also easier to administer. Mouse-tracking was chosen as the process-tracing method for the “Dira” task, also because it allows running several studies in parallel in a non-invasive setup using standard hardware participants are familiar with.

## 3. Methods

### 3.1. Experimental design and conditions

The computer-based experiment “Dira” is programmed as a series of different screens. From the participants' perspective, “Dira” combines perceived freedom to explore the task with aesthetically pleasant stimuli. Participants interact with the text and images of the task by hovering the mouse pointer over these elements. The order and duration of these interactions are up to the prospective problem solvers. The images are taken from the “Dixit” card game which has been praised for its artistic and beautiful drawings. Moreover, the whole experiment is designed like a game. These design choices are intended to make the “Dira” tasks “inherently interesting or enjoyable,” one of the critical elements that are known to increase intrinsic motivation in participants (Ryan and Deci, 2000, p. 55). In turn, Baas et al. (2008) and da Costa et al. (2015) have shown positive correlations between intrinsic motivation and performance in creative problem solving tasks.

For the current study, “Dira” was administered in three different between-subject conditions. In condition 1 “Dira” does not provide any feedback and participants have no reference to evaluate their answers and performance in the task. In condition 2 we added a potential solution to trigger extrinsic insights. Given that tasks are often perceived as difficult, this demonstrates a possible solution to the participants and hence is thought to increase the motivation to solve the next problem. Furthermore, these solutions have the potential of triggering extrinsic insights, which are a special type of insight following the recent argument by Rothmaler et al. (2017). Given the correlation between mood and insight (Subramaniam et al., 2009; Akbari Chermahini and Hommel, 2012) a triggered Eureka experience could have a positive effect on the intrinsic motivation and metacognition. In condition 2 we want to explore if this leads to a change in the reported experience and observed behavior. In condition 3 we ask participants to elaborate on their reported solution. We expect this verbalization of an answer to increase the metacognitive awareness during task execution (Hedne et al., 2016) and hence an effect on “quiz time” and reported Eureka experience. Condition 1 was the first to be run and all participants at the time followed the same protocol. Subsequent participants at a later time were randomly assigned to either condition 2 or condition 3.

**Figure 2:**
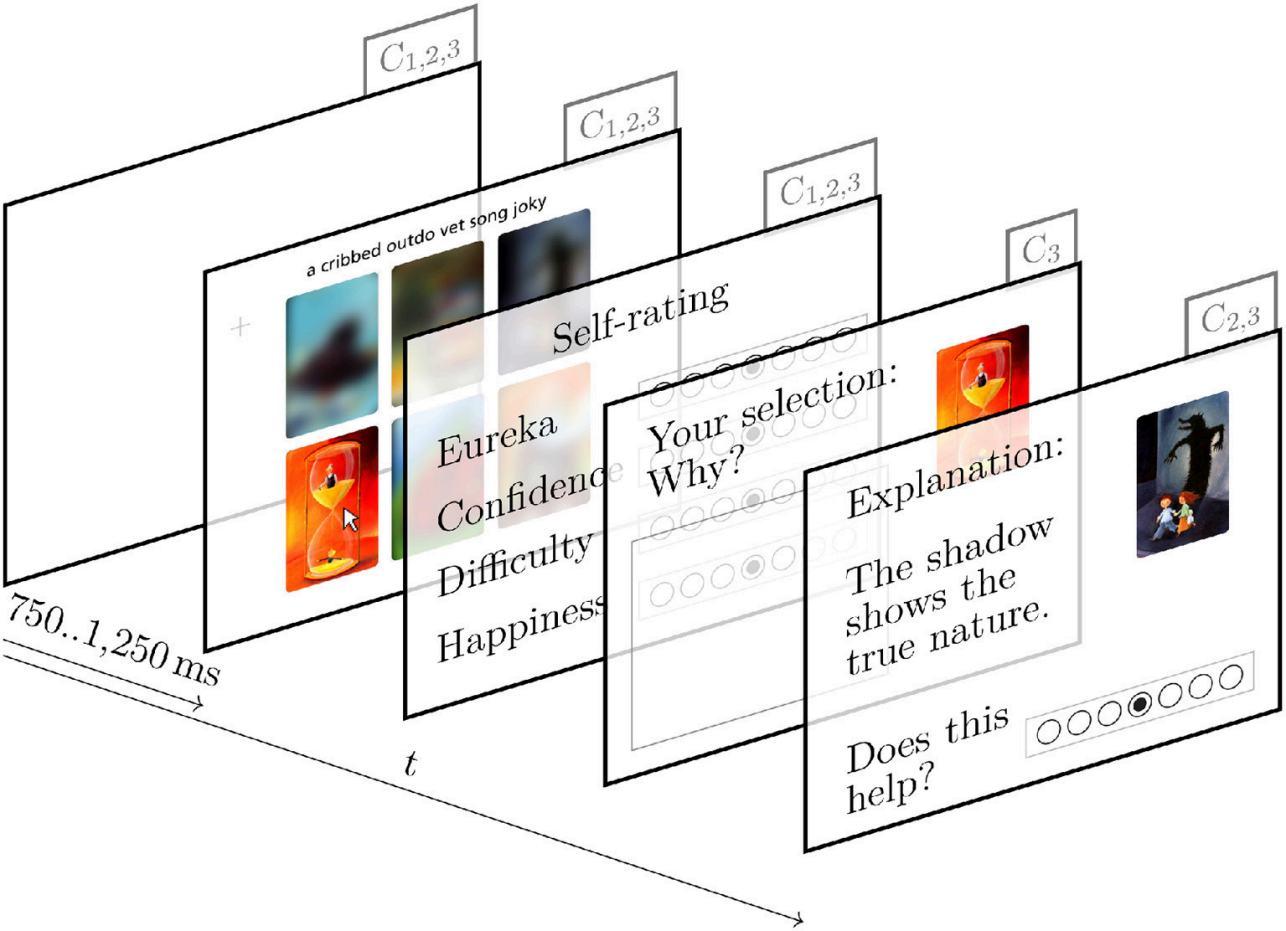
Experimental conditions of the “Dira” rounds. Each parallelogram represents a screen and the annotation in the right upper corner identifies in which condition the screens are used.

In condition 2 the additional screen “explanation” is added to each round as illustrated in Figure 2. Appended after the “rating,” it is the last screen before the start of the next round. The “explanation” screen shows the “intended solution,” the image that initially inspired the storyteller to invent the text. We also show a short explanation on how the intended solution and text are connected. The short sentence is based on a text taken from the stimulus dataset and is designed to help the participants: One method to solve a “Dira” task is to empathize with the storyteller and find the intended solution that initially inspired the text. To assess the success of this help, we then ask the participants to rate “How much does the Explanation help [you] to understand the association between image and text?” Their answer ranges from “not at all” to “very much” on a seven-point Likert item. Submitting the answer starts the next round of condition 2 with a “fixation cross.”

In condition 3 an “elaboration” screen is placed between the “rating” and the “explanation” screen as shown in Figure 2. In this screen, participants see the given text and their selected image, and they are asked to elaborate on their decision. Afterwards, they see the same “explanation” screen as described above. Once they have completed these additional screens, participants restart the next “round” of condition 3 with a “fixation cross.”

### 3.2. Procedure

Any “Dira” experiment starts with an opening sequence consisting of a “welcome” screen, a “questionnaire,” and a “description” of the task. This initial series is followed by 40 rounds containing a “fixation cross,” “quiz,” “rating,” and optional “explanation” or “elaboration” screens. The experiment concludes with an on-screen “debrief.”

A “welcome” screen explains the basic idea of the study as well as potential risks and the right to withdraw data. The study only continues if participants understand and agree to the minimum requirements that have been cleared by the Faculty of Health and Human Sciences Ethics Committee at Plymouth University. Once participants have given their consent, they are shown the “questionnaire.”

During the “questionnaire” participants are asked to specify their age, gender and primary language and if they have participated in the study “Dira” before. They are also asked to rate their fluency in understanding written English and familiarity with the card game “Dixit” on a seven-point Likert item. Participants are also asked to rate themselves in 14 additional seven-point Likert item questions, four of which belong to the Subjective Happiness Scale (SHS) developed by Lyubomirsky and Lepper (1999) and ten more of the Curiosity and Exploration Inventory II (CEI-II) as published by Kashdan et al. (2009). The scales were chosen because emotional states (Baas et al., 2008), openness to experience, and intrinsic motivation (Eccles and Wigfield, 2002) are known to influence problem solving (Beaty et al., 2014). These results are not discussed here since the interaction between individual differences and the performance in the “Dira” task are beyond the scope of the current article.

Once participants have completed the questionnaire, the procedure of the experiment is explained to them in detail in a “description” screen. This screen also holds a minimal and neo-Gestalt inspired definition of the “Eureka moment” as “the common human experience of suddenly understanding a previously incomprehensible problem or concept,” for accessibility reasons taken from Wikipedia (2016). Afterwards, the 40 “rounds” of the experiment begin.

Each “round” starts with a “fixation cross” which is shown at the center of the screen for a randomized time between 750 and 1,250 ms. Afterwards text and images appear on the “quiz” screen as illustrated in Figure 1: one text on top and six images in a grid of two rows by three columns. Unless the participants hover the mouse on top of these elements, the letters of the text are shown in a randomized order, and the images are strongly blurred. An example can be seen in the second screen of Figure 2 which shows the text “Don't judge a book by its cover” with the letters in a randomized order and images blurred except for “image f” over which the mouse pointer hovers. The recording of hover times during the “quiz” allows to track when participants pay attention to each of the elements and for how long (Navalpakkam and Churchill, 2012). On this screen, participants attempt to find the image that they think is most likely associated with the text and select it through a single click. There is no time limit for completing this task. Once participants have chosen a solution, they advance to the “rating” screen.

During the “rating” screen, participants are asked to rate their performance in the “quiz.” They are asked the following four questions, with the range of possible responses on seven-point Likert items in brackets: “How confident are you that the solution is right?” (not confident—very confident), “How hard was it for you to come up with the solution?” (not hard—very hard), “How strong did you experience a Eureka moment?” (not at all—very strong), and “How happy are you with your answer?” (very unhappy—very happy). After submitting the answers, the next round starts with a “fixation cross.”

Participants who have completed the 40 rounds conclude their participation with the “debrief” screen. Here they are informed that the study intended to measure the timing of their behavior during the “quiz.” Participants are encouraged to give additional feedback concerning the experiment, and they have the option to leave an email address in case they want to be informed of the results of the study. This on-screen debrief was followed by a short unstructured personal discussion relating to their experience in the Dira experiment.

### 3.3. Task administration

The controlled study “Dira” was designed as a computer-based task administered in a laboratory setup. The task was delivered through a custom developed web application delivered through a full-screen web browser. The same type of computer mouse with an optical sensor and the same type of 22 inch LCD screen with 1,920 × 1,080 pixel resolution were used for the whole experiment. Participants are most likely familiar with the setup as it is the same hardware available to students in library and public computing spaces across campus. The experiment was delivered in a dedicated room with no more than five participants at the same time who were asked to stay silent during the experiment. Welcome and debrief was performed outside the room to keep any distraction to a minimum. Informed consent was collected from participants; then they were accommodated at a computer showing a “welcome” screen.

### 3.4. Participants

One hundred and twenty-four participants between the age of 18 and 56 ($\bar{age}=22.6$, *sd* = 6.99) were recruited from a local pool of pre-registered psychology students and a second pool that was open to students of other courses and members of the public. While two of the participants chose not to report their gender, 83 identified as female and 39 as male. Psychology students received course credits and points for running their studies. Participants from the second pool received monetary compensation. The overall sample appears similar to the one described by Henrich et al. (2010).

### 3.5. Data pre-processing

The data collected during the “quiz” of the “Dira” task are intended to trace the participants' thought process through their behavior. The recorded dataset includes chronological information concerning the order in which participants engage with elements, as well as the duration of the interactions.

The chronology or order in which participants engage with elements shows that they do not interact with all elements in each round. If participants do not look at the text, this has implications on their ability to solve the problem: Participants who have not seen the text will not be able to find an association between the text and one of the images for this particular round. On the other hand, if they have seen the text but not all images, they are still able to find a solution. Rounds in which participants did not look at the text were therefore excluded from further analysis, whereas rounds with missing interactions for some images were still analyzed. Furthermore, cognitive processes deployed in rounds that start with the text might differ from the ones starting with one of the images. To control for these different modalities, we focus in this paper on the rounds starting with text and remove all others.

**Figure 3:**
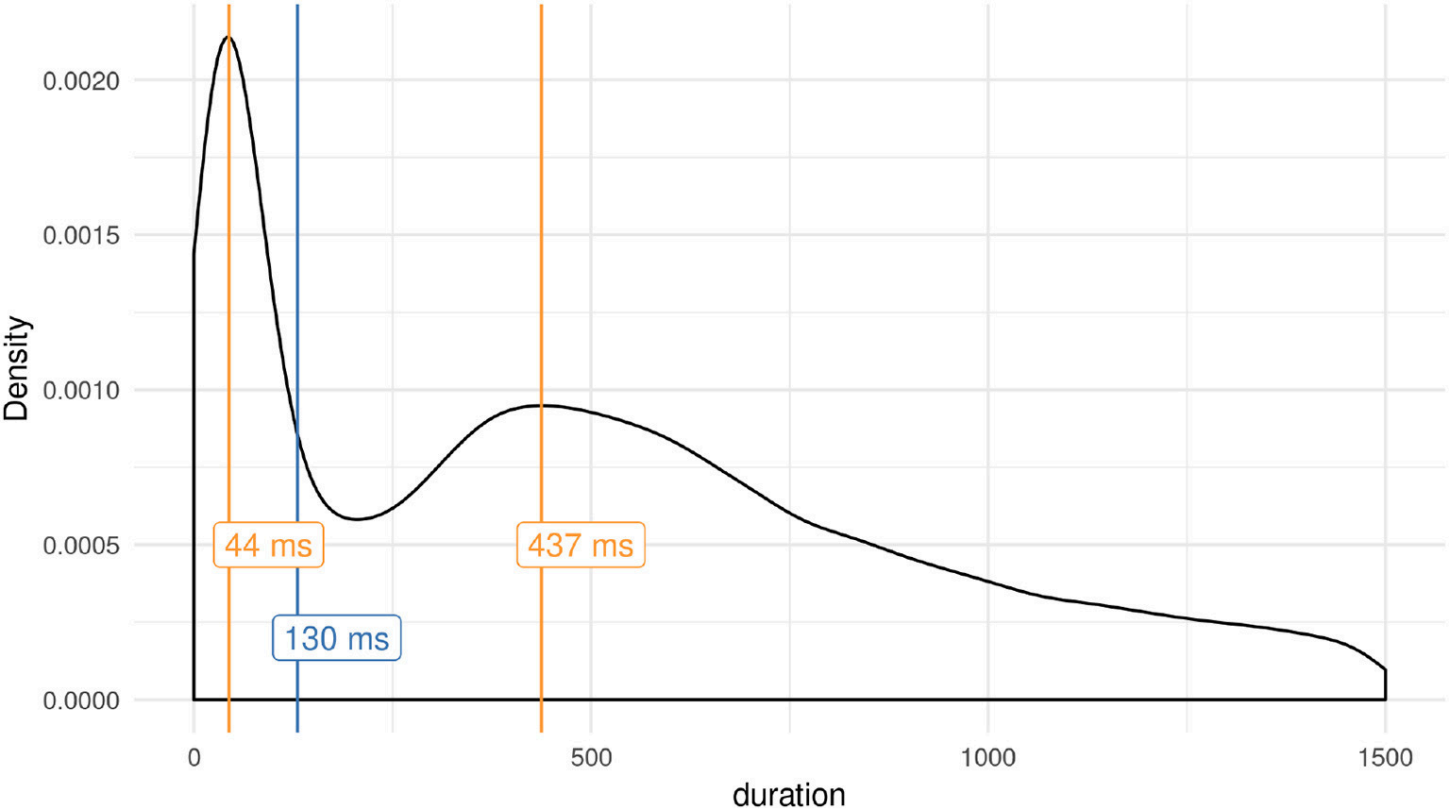
Distribution of hovering times on elements during the “quiz.” The modes of the bimodal distribution are marked with red lines. The cutoff time between the two distributions, a result of the classification described in the text, is shown in blue.

**Figure 4:**
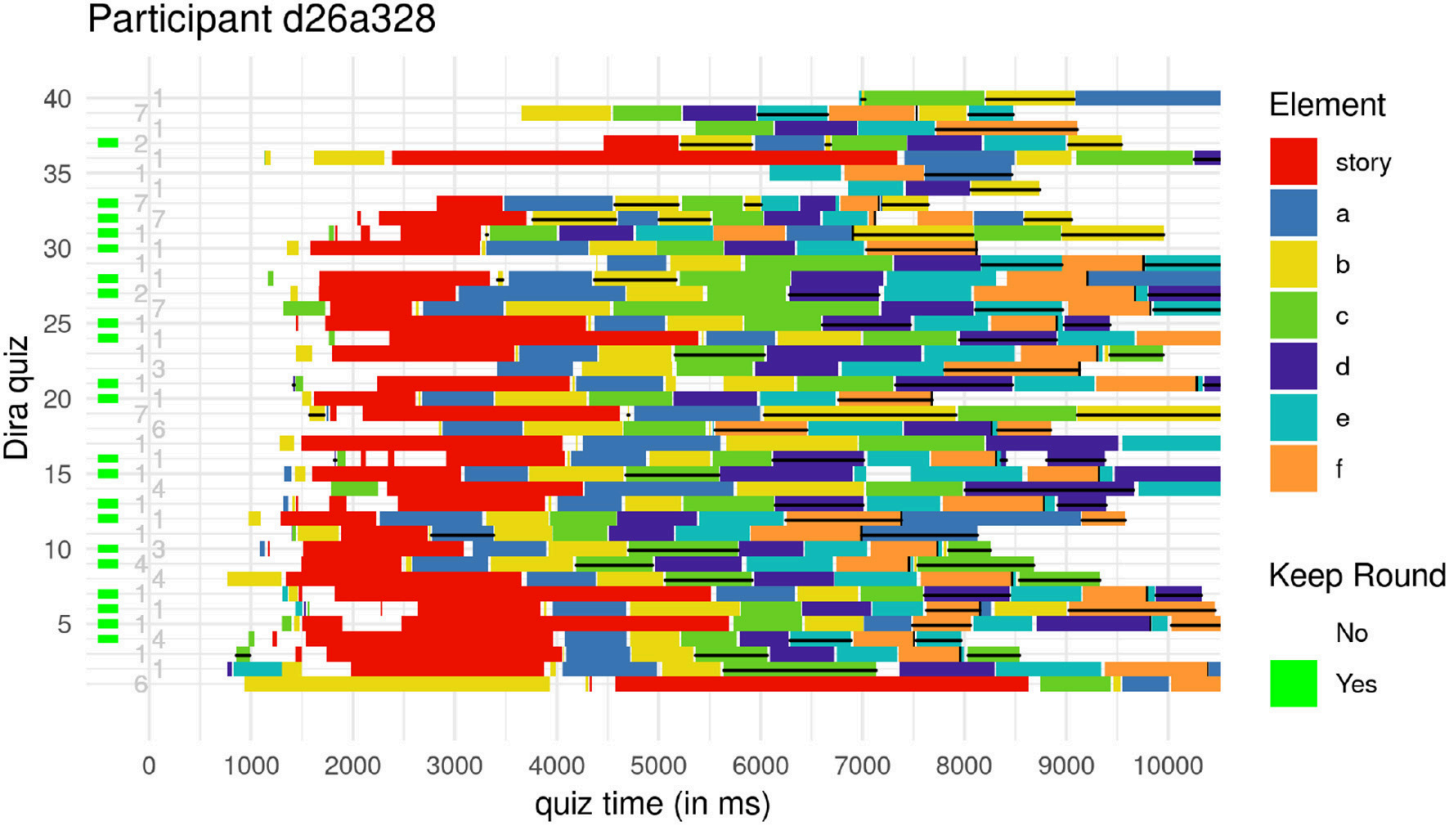
One participant's interaction with text and images during the first 10 s (x-axis) of each of the 40 rounds (y-axis). The length of each colored bar notifies the duration, the color identifies the position of the element. Horizontal black lines mark the items that are selected in this round, the vertical black lines mark the end of the “First Full Scan.” The numbers between one and seven next to the y-axis show the reported strength of the Eureka experience for that round. The green blocks mark rounds that are kept for further analysis. For details see text.

The duration of interactions with text and images is assumed to relate to the amount of acquired and processed information. However, the data also include quick movements that do not contribute to acquiring information, as illustrated in Figure 3. If people want to look at an element not adjacent to the current mouse position, they need to move the pointer across one or more elements. In this case, the distance of the mouse pointer from the target image is between 1.5 times and 4.3 times the size of the target. According to Fitts' law, the task of moving to a distant image has an index of difficulty between 1.3 and 2.4. Applying the extreme values for throughput suggested in Soukoreff and MacKenzie (2004), participants are estimated to require between 260 and 640 ms for the whole distance and therefore between 150 and 170 ms to cross an image between the starting position and the target image. During this movement, the element is briefly unblurred on screen. Figure 4 shows examples of this movement at the beginning of rounds 4–7. The density of the duration of interactions in Figure 3 shows how often participants interact with elements for certain durations. The bimodal distribution suggests that there are at least two different types of behavior recorded. Shorter interactions, in Figure 3 marked as the local maxima around 44 ms, are distinctly different from longer hover times peaking around 437 ms. A cluster model fitted to the log-transformed duration using two components (Scrucca et al., 2016) classifies 17,849 interactions as short and 63,452 as long, divided at 130 ms. The predicted movement time according to Fitts' law and the identified time dividing the bimodal distribution of hover times suggest that the shorter engagements with elements might be movements across the element, targeting another one. If participants follow the mouse movement and see the intermediately unblurred image on screen during the shorter engagement, the following unblurred target image acts as a backward mask. Previous research does not provide evidence for perceptual discrimination between visual stimuli shown for less than 100 ms (VanRullen and Thorpe, 2001; Zoefel and VanRullen, 2017). Furthermore, Salti et al. (2015) argue for a required exposure of more than 250 ms necessary to consciously perceive a stimulus. Assuming that specific information from a higher conceptual level is required to identify remote associations in the “Dira” task, these activations would require additional time, as Quiroga et al. (2008) have shown in single neuron recordings. For the “Dira” experiment we are interested in interactions for which participants can distinguish between different images. Concluding the different cited streams of research we assume that shorter interactions from the bimodal distribution shown in Figure 3 have no or little influence on the process “Dira” intends to capture. In accordance with Fitts' law, we assume that the shorter observed behavior represents mouse movements across elements moving for a different target without cognitive processing of the image. Consequently, element interactions below the identified 130 ms are excluded from further analysis.

## 4. Results

We first report on the type of raw behavioral data collected during the “quiz” and derived measures such as the chronology of information acquisition. Secondly we present the self-reported measures collected during the “rating” screen. We then show that the number of interactions with elements relates to the reported strength of the Eureka experience. Finally, we report results of the length of different interactions in comparison to the reported strength of reported Eureka experience. For the statistical tests we adopted a critical α level of 0.01 as originally put forward by Melton (1962) and Trafimow et al. (2018). For each test where the estimated amount of false discoveries surpasses this threshold, we transparently report this value as suggested by Lakens et al. (2018). We adopt this practice for our study and the chosen traditional threshold, in particular since the discussion on statistical testing is far from over (Benjamin et al., 2017; Trafimow et al., 2018).

### 4.1. Available process-tracing measures

Participants' interaction with elements on the “quiz” screen is a metric for tracing their problem solving process. The time to produce solutions has previously been used in convergent thinking tasks (Salvi et al., 2016) and divergent thinking tasks (Forthmann et al., 2017), a measure that is similar to the “quiz time” in this paper. “Dira” employs a novel method by collecting behavioral data, namely the interaction times with the stimuli, throughout the creative process. This is a novel approach by shifting the focus from measuring the duration to produce a “creative product” to providing chronological measures of the process itself. While the current paper focuses on the moment solutions emerge, the experimental paradigm could be used to trace other aspects of the creative process such as preparing for the task or the verification of solutions. Since the extracted behavioral measures are vital for understanding the subsequent writing, we elaborate on the raw data and their derived measures in this section.

To illustrate the kind of data collected in “Dira,” we will now discuss in detail Figure 4. The duration of interaction with each element is the difference between offset and onset time which is the raw data recorded during the task. Figure 4 shows the example of one participant's interaction within the first 10 seconds of each of the 40 rounds. Each of the colored bars represents a timespan during which the mouse pointer hovers on top of an element. The length represents the duration, and the color signifies with which element the participants interact. For example, in the first round on the bottom of Figure 4, this particular participant spent a long time on “image b” (for color and naming scheme see Figure 1). The second round instead starts with three short interactions with “image d,” “image e,” and “image b” followed by a short time without any element interaction before hovering on top of the “text” for almost two seconds. Some rounds, like the third one, are finished within the ten second period shown in Figure 4, others like the first two continued for a more extended period.

Figure 4 also shows additional data that is available in “Dira.” We refer to the moment participants select their solution as the “quiz time” since it ends the current “quiz.” This measure is similar to existing measures in other tasks, such as the total time to solve convergent thinking tasks as reported by Salvi et al. (2016) or to produce utterances for divergent thinking tasks (Forthmann et al., 2017). The example participant selects the solution for round 3 at around 8,500 ms and round 4 at around 8,000 ms. The selected solution, for example, “image c” for round 3, is also indicated as a horizontal black line for the rounds in Figure 4. The vertical black line marks the end of what we call the “First Full Scan,” the end of the interaction with the seventh unique element. Participants have interacted with each element at least once at the end of the “First Full Scan.” The number next to the vertical axis in Figure 4 represents the strength of the Eureka moment participants indicate during the “rating” screen. The example participant had no Eureka experience in round 2 and 3, but a strong one in round 19 and 26. Finally, the green box next to the vertical axis indicates rounds that are part of the analysis and not filtered out for one of the reasons explicated previously.

We administered “Dira” in three different conditions with a between-subject design as introduced in section 3.1. Based on the previously provided argument we hypothesized a longer interaction time for conditions 2 and 3. To test this, we built two linear mixed-effects models. Firstly we used the length of the First Full Scan as a dependent variable with the participant and round of the experiment as a random effect. We found no evidence for a difference between the three conditions (χ^2^(2) = 2.4, *p* = 0.3). In a second model, we used the quiz time as the dependent variable as it is most similar to the task time used in other tasks (Salvi et al., 2016; Forthmann et al., 2017). With participant and round of the experiment as random effects, we found no evidence that would support an effect of the experimental condition on time to report a solution (χ^2^(2) = 0.87, *p* = 0.65). Without support for the effect of the experimental conditions, there is no argument to distinguish between the three conditions regarding behavioral data.

### 4.2. Available self-reported measures

Participants in the “Dira” task are required to provide self-reported measures in addition to the implicit behavioral data collected during the “quiz.” During the “reporting” screen they are asked to account for the strength of their just encountered Eureka experience, their confidence in the given solution, the perceived difficulty of the task, and their current happiness on seven-point Likert items respectively. Besides, participants in condition 2 and 3 are also asked to rate how well they understand the connection between the text and a potential solution. In condition 3 they are furthermore asked to write down how their solution is associated with the text. These measures are collected during each of the 40 rounds. In section 3.1 we hypothesized an increase in the reported Eureka experience for condition 3. Nevertheless, this is not supported by the collected data (χ^2^(2) = 4.81, *p* = 0.09). Consequently, we cannot maintain a separate analysis for the self-reports in the three conditions.

**Figure 5:**
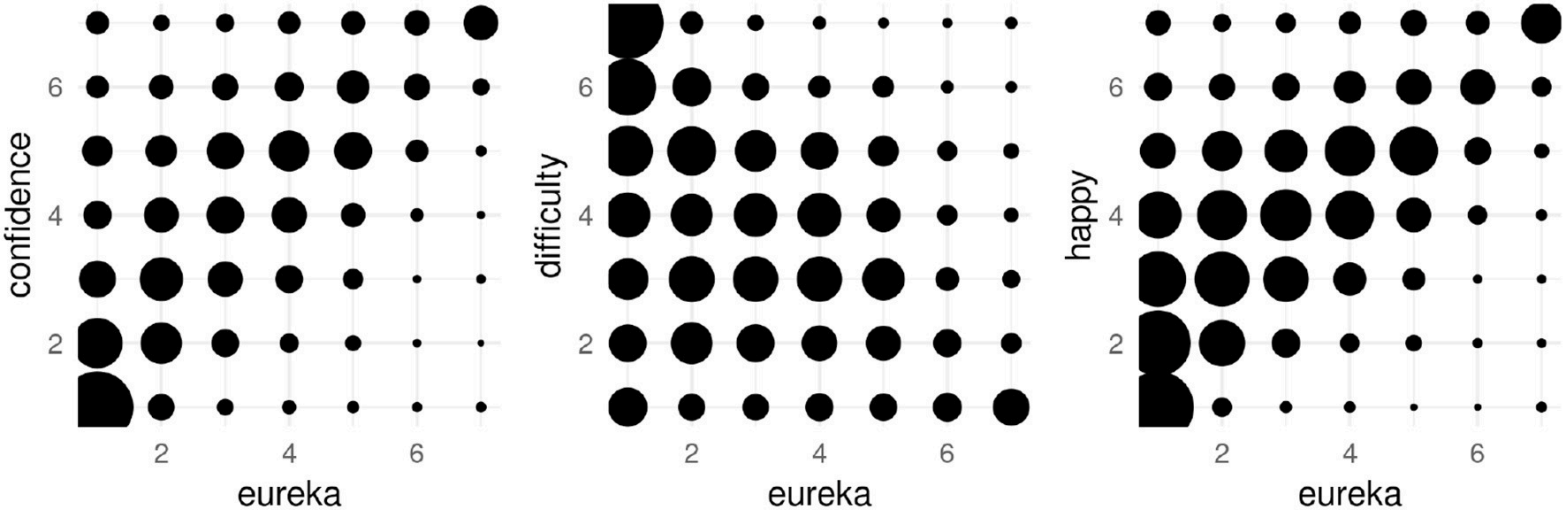
Confidence, perceived task difficulty, and happiness related to the reported strength of the Eureka experience. The size of the circle represents the number of rounds in which the combination was reported, larger circles representing more answers.

As illustrated in Figure 5, for rounds in which participants report a strong Eureka experience they are also confident regarding their solution. Rounds with weaker or no Eureka experience are reported across the whole spectrum of confidence, but with a tendency toward low confidence as well. Instead, rounds with strong Eureka experiences are rarely rated as low confidence. This asymmetry leads to an overall Spearman's rank correlation of ρ = 0.62, *p* < 0.01. In contrast, rounds with strong reported Eurekas rank low in difficulty and rarely as “hard to come up with a solution.” Rounds with a low or no Eureka experience are perceived with varying difficulty. The overall correlation between the reported Eureka experience and stated task difficulty is ρ = −0.41, *p* < 0.01. Finally, for weak or no perceived Eureka, participants express a range of different happiness, but only high happiness for strong Eureka experiences. Reported Eureka and happiness are correlated by ρ = 0.6, *p* < 0.01. The reliability of the rating is either good for reported Eureka (α = 0.86) and difficulty (α = 0.87), or acceptable for happiness (α = 0.78) and confidence (α = 0.77) based on Cronbach's alpha. Conceptually these four measures are linked by the literature review of Topolinski and Reber (2010a), who discuss the relationship between ease, positive affect, and confidence to insight. This link is reflected by the data collected in “Dira” with good reliability suggested by Cronbach's α = 0.86 across the four measures. Consequently, these findings confirm our second hypothesis that participants can report their experience on more than a binary scale.

### 4.3. Number of interactions

**Figure 6:**
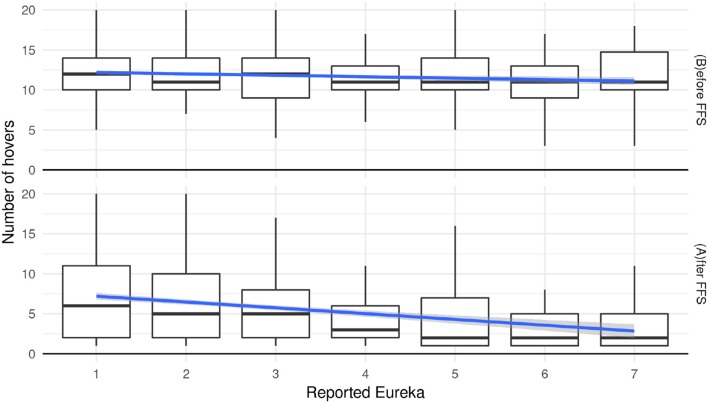
Number of hovers before **(Top row)** and after **(Bottom row)** “First Full Scan” over the reported strength of Eureka experience.

In this section, we take a first look at the relationship between the self-reported intensity of the Eureka experience and the chronology extracted from the behavioral data. For example, when participants acquire information during the “quiz” and they find a solution, they might stop looking at more images. Therefore we hypothesize that the Eureka experience is stronger for rounds with fewer interactions. Figure 6 shows how many elements a participant interacts with during each of the 40 rounds of the “Dira” experiment. The sub-figure on the top shows the number of interactions during the “First Full Scan” before participants have seen each element at least once. An average of ten to twelve interactions means that participants tend to go back and forth between elements even before they have seen all seven elements. More specifically, if participants look at elements in a certain order, looking back at one element and then continuing with the round can result in two additional interactions. To give an example: one participant has looked at “image a” and “image b” and then goes back to “image a” before continuing with “image b,” “image c,” and “image d.” In this case, the participant had interacted twice with “image a” and “image b” during the “First Full Scan.” This particular round would have accounted for at least nine interactions before the end of the “First Full Scan.” To arrive at the numbers shown in Figure 6, this seems to happen twice in a typical “First Full Scan.”

To test the above hypothesis, we built an ordinal mixed-effects model (Christensen, 2015) with reported Eureka as a dependent variable. The number of interactions, the classification into before and after “First Full Scan,” and the experimental conditions were used as predictors. The rounds of the experiment as well as participants were considered as random effects. Results from this model indicate that there is a significant negative effect (estimate = −0.06, *z* = −6.27, *p* < 0.01) of numbers of hovers on the reported Eureka before the end of the “First Full Scan.” The model also shows a significant negative effect (estimate = −0.35, *z* = −3.68, *p* < 0.01) for the number of interactions after the end of the “First Full Scan.” This confirms our hypothesis for the interactions during and after the “First Full Scan.” On the other hand, there is no evidence that condition 2 or 3 have an effect compared to participants in condition 1 (estimates = [−0.12, −0.28], *z* = [−0.35, −0.88], *p* = [0.73, 0.38]).

During the “First Full Scan,” the above model shows a significant effect of the number of interactions with elements on the strength of the Eureka experience. Across all conditions, this difference is between 12.61 interactions for no or low Eureka experiences and 11.38 interactions for strong reported Eurekas. After the “First Full Scan” participants do not interact with all the images and text, again. The significant effect of the number of interactions on the reported strength of Eureka is higher this time and more pronounced in Figure 6: the difference is between 9.65 interactions for no experience of a Eureka and 4.24 interactions for a strong one. There is no evidence for an effect of the experimental condition on these results. Considering that the behavior of participants with different Eureka experiences seems to change before the end of the “First Full Scan,” it is of interest to examine the behavior during the “First Full Scan” in more detail. Hereafter we will examine whether the duration of hovering over elements provides additional information.

### 4.4. Last hover during first full scan

**Figure 7:**
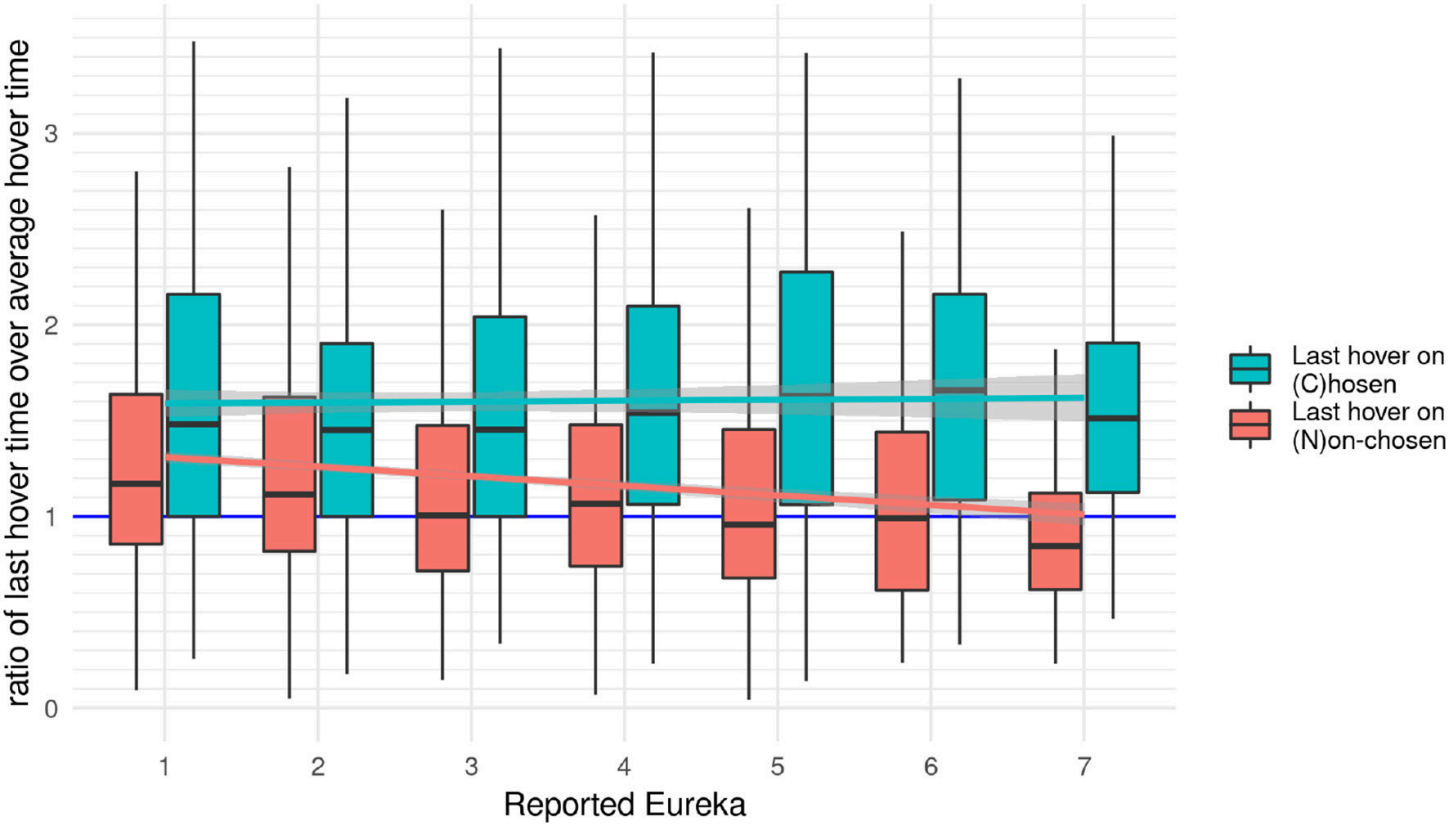
The ratio between the last hover time within the First Full Scan and the average of all other hover times in the “First Full Scan,” separated by rounds in which participants hover over the chosen image last vs. the ones they look at another picture. A value of one means that they are equal, lower than one means the last scan is shorter than the previous ones. In addition to the box-whisker plot (showing the median and distribution), the lines show a linear model fitted to the mean ratio and surrounded by the 95% confidence interval in light gray.

Here we report the results for the hover duration on the seventh unique element. It is the last image during the “First Full Scan” and the first time participants interact with this specific element. Following up on the previous finding of an interesting difference between interactions during and after the “First Full Scan,” we want to narrow down the time of emerging solutions by exploring this specific hover time. More specifically we show the ratio of the duration on the last image compared to the mean of previous interactions. The chronometrical measure of hover time is illustrated in Figure 7. To correct for individual differences in processing speed, we plot the ratio of the hover time on the last image and the average hover times on all other images during the “First Full Scan.” Figure 7 plots separately the ratio of rounds in which this element is the one (C)hosen later in the experiment and rounds which end on a (N)on-chosen one.

Figure 7 shows two effects: Firstly, for the “First Full Scans” ending on a chosen image, the median of the hover time is roughly 50% higher on that element than for non-chosen ones (1,323 vs. 855.9 ms). Secondly, less time seems to be spent on the last non-chosen image than on the previous ones for stronger Eureka experiences, whereas more time is spent on the last image for low Eureka values. To quantify these effects we built an ordinal mixed-effect regression model with the strength of the reported Eureka experience as a dependent variable and the ratio, the type of element for the last hover, and the experimental condition as predictors. The round of the experiment and the participant were used as random effects. This model shows a significant effect of the ratio on the strength of the reported Eureka (estimate = −0.24, *z* = −6.1, *p* < 0.01). It further shows a significant effect for rounds in which the last element is the chosen one on the strength of the reported Eureka (estimate = 0.2, *z* = 2.71, *p* < 0.01). There is no evidence for the ratio in condition 2 or 3 affecting the reported Eureka intensity (estimate = [−0.09, −0.55], *z* = [−0.32, −0.32], *p* = [0.75, 0.05]).

The negative slope of the ratio over the strength of Eureka, in Figure 7 particularly evident for the last hover on the non-chosen image, suggests that a solution has emerged before the end of the “First Full Scan.” The change of the ratio is either the result of a decrease of the numerator, an increase of the denominator, or a combination of both. The numerator decreases if participants spent less time on the last image when having a stronger Eureka experience. The denominator represents the average time spent on all previous images. It increases if participants spend more time on at least one of the previous images. If participants had Eureka experiences while looking at the image they are going to choose later, and this would be associated with them looking longer at that image, this would increase the denominator in the rounds which end on the non-chosen images. The observed increase would also explain the difference between rounds that end on chosen and non-chosen images. If participants spent less time on subsequent images, for example after a Eureka experience, this would decrease the numerator for the rounds ending on non-chosen images, but not for the ones ending on the chosen images. This interpretation of the observations suggests that the measured ratio is a compound of chronological effects and hover duration. Therefore we focus now on the duration spent on the chosen image and its relation to the strength of Eureka.

### 4.5. Chosen images and length of interactions

**Figure 8:**
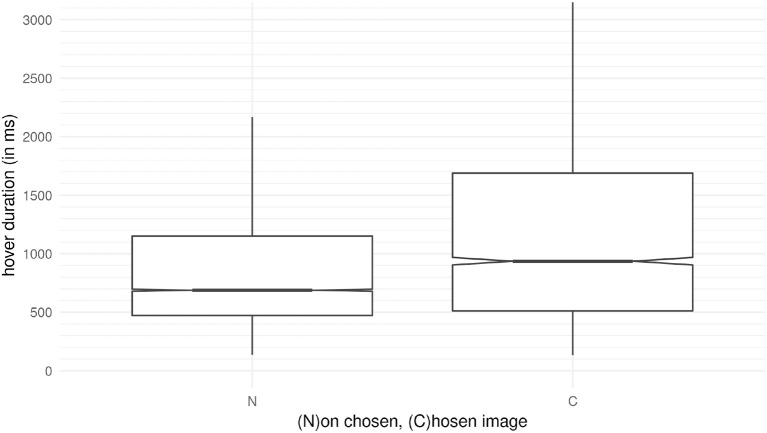
The hover duration on the images within the First Full Scan. The time on the (C)hosen picture is longer the time on the five other images that are (N)ot chosen.

The observation of the ratio of interaction times during the “First Full Scan” suggests that the interaction times between chosen and non-chosen images differ. Instead of a compound measure, we purely show the duration of hover times during the “First Full Scan” on (C)hosen and (N)on-chosen images in Figure 8. A Mann-Whitney test indicates that the duration of viewing chosen images (duration = 935.9 ms) is significantly longer than for non-chosen pictures (duration = 687.8 ms), *U* = 20,873,370, *p* < 0.01). Furthermore, there is a significant difference between the three conditions regarding the hover duration on non-chosen images (*H* = 42.07), *p* < 0.01 *Md*_Condition 1_ = 663.2, *Md*_Condition 2_ = 679.7, *Md*_Condition 3_ = 727.9), according to a Kruskal-Wallis test. Furthermore, there is a difference between conditions for the chosen images (*H* = 9.18, *p* = 0.01, *Md*_Condition 1_ = 879.8, *Md*_Condition 2_ = 915.9, *Md*_Condition 3_ = 1,048). Participants spend a significantly longer time on the chosen image in the third condition than in the other two conditions, and more time in the second condition compared to the first one.

We now look at the link between hover duration and reported Eureka experience in more detail. We built an ordinal regression model with the reported strength of the Eureka experience as the dependent variable. With the hovering time on the chosen images as a predictor, we failed to find evidence for a link between the strength of the Eureka and interaction time (estimate = 0.01, *z* = 0.21, *p* = 0.83). This is not unexpected since the raw data include slower and faster participants. Instead, if an ordinal mixed-effects model considers the participant as a random effect, the evidence supporting the link between hover duration and Eureka experience surpasses the threshold (estimate = 0.14, *z* = 3.16, *p* < 0.01). From this example we conclude that the recorded raw hover durations with text and images have little validity in connection with the self-reported measures collected during the “rating” screen. To address this, we remove the influence of participants and the task by considering the ratio between the time spent on chosen and non-chosen images calculated separately for each round. This suggested ratio between interaction times for a single round and with a single participant does not include chronological components related to the order of interactions; it is between measured times only.

**Figure 9:**
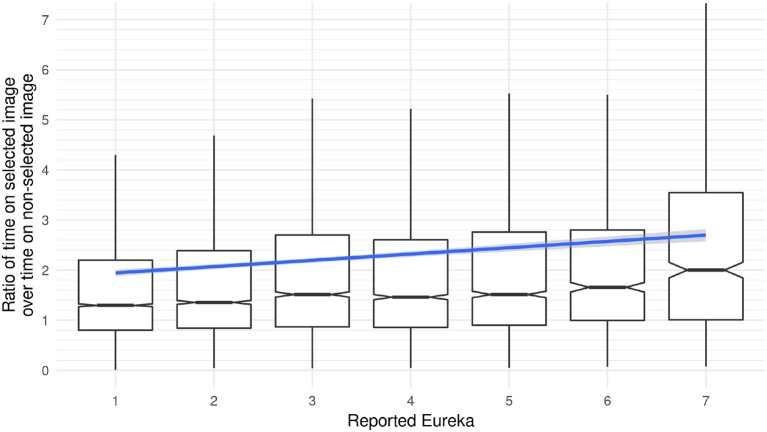
The ratio of time spent on the chosen image over non-chosen images as a box-whisker plot with a linear model fit to the mean. The median for the ratio depicted in the box-whisker plot shows that participants spend nearly 1.3 times as much time on the chosen image compared to the others for a low Eureka, but about twice the time for a strong Eureka. The difference between mean (linear model) and median (box-whisker) results from outliers in the data.

Figure 9 shows the ratio between the hover duration on the chosen image and the average time spent on the other images. This ratio is higher for rounds in which participants report a stronger Eureka experience. An ordinal mixed-effects model fitted to the data supports this observation. The model uses the strength of the reported Eureka experience as a dependent variable and the ratio between the time spent on the selected image compared to the average duration on all other images as well as the experimental condition as a predictor. The round of the “Dira” task and the participant are used as random variables. This model confirms that an increase in the ratio corresponds to a stronger Eureka experience (estimate = 0.02, *z* = 5.65, *p* < 0.01). With a ratio of 1.3 for no Eureka and 2 for a strong Eureka, participants seem to spend approximately 50% more time on the chosen image in rounds when they report a strong Eureka experience. However, the model does not provide evidence for an influence of condition 2 or 3 on the reported Eureka (estimates = [−0.1, −0.58], *z* = [−0.33, −2], *p* = [0.74, 0.05]).

Here we have presented two main findings. Firstly, the observations of the length of interaction with elements show that participants spend more time on the images they will select later in the task. Secondly, for rounds with a strong reported Eureka experience, the time spent on the chosen image is significantly longer than in rounds with a weaker or no Eureka experience.

## 5. Discussion

The moment when a solution to a problem emerges is an extraordinary experience. It causes people to cry out “Eureka” (Pollio, 1914), “Aha” (Bühler, 1908), or “Uh-oh” (Hill and Kemp, 2016) and often their mood increases. In this paper, we suggest “Dira” as a novel experimental paradigm to observe these moments as part of the creative process. Many previous studies rely on the judgement of creative products, persons, or press (Rhodes, 1961)—or use proxy phenomena to assess the process contributing to creativity, innovation, and problem solving. In this study, we tested 124 people who participated in a controlled lab experiment designed to study the emergence of solutions. “Dira” records behavioral data during each task to observe the creative process directly. Specifically, we determine the chronology and chronometric measures of participants' interaction with potential solutions. After each task, we ask the participants to self-report their experience on four different items. Here we discuss the implications of combined behavioral and metacognitive measures in the “Dira” task.

### 5.1. Eureka experiences in “dira”

Results from the behavioral data within the “First Full Scan” of “Dira” show that participants spend longer times on images they are going to select as their solution. Moreover, the length of the interaction on these chosen images is linked to the strength of the reported Eureka experience, with longer hover durations associated with stronger Eureka experiences. As shown in section 4.4, the median interaction time on the chosen image is about 50% longer than on the non-chosen ones. Another result related to the strength of Eureka is reported in section 4.5. For rounds that evoke a strong Eureka experience, participants spend about 50% more time hovering on the chosen image as compared to rounds with no or low reported Eurekas. The current analysis does not allow drawing any conclusions regarding causality. Future studies could test if more extended engagement yields stronger Eureka experiences or if stronger Eureka experiences lead to longer hover durations.

After participants have interacted with the chosen image, they are less likely to continue looking for more elements according to the results in section 4.3. Supposedly participants continuously scan the elements on the screen for a solution. If they find an association, the number of elements they interact with afterwards is related to the strength of the Eureka experience reported later. The significant effect can be observed as early as during the “First Full Scan” and the initial interaction with the images. These results suggest that something distinctive might already be happening during the initial engagement with the images.

With support from the ordinal mixed-effects model considering behavioral and self-reported measures, we confirm our first hypothesis that behavior happening during the “quiz” results in the reported intensity of Eureka. It would seem natural that the Eureka experience also happens during this time. However, it is not impossible that the Eureka experience is the result of a post-event evaluation. In any case, due to the short quiz time, these experiences would qualify as immediate insights according to Cranford and Moss (2012). In their study of convergent thinking, they found a difference between solutions found through a “classical insight” sequence and “immediate insights.” The immediate insights only consisted of an “Aha!” or Eureka experience and were considerably faster. This quick insight is also in line with the idea of intrapersonal creativity or mini-c introduced by Beghetto and Kaufman (2007). It would be interesting to design a modified version of “Dira” to elicit non-immediate insights as well, for example by tapping into the thought suppression as used in the delayed incubation paradigm (Gilhooly et al., 2014) or more generally in “little-c” type of tasks. We leave this speculation for future studies.

### 5.2. Subjective experience

In more detail, the strong Eureka experience in rounds with high confidence is consistent with previous findings, for example by Hedne et al. (2016). In their study on magic tricks, problems solved via insight were rated with higher confidence than problems solved without insight. Previously Danek et al. (2014) had assessed a higher confidence rating for insight solutions as well, but they had used confidence in the definition of insight given to the participants, so this could have been a potential confound in their results. Hedne et al. (2016) also explicitly link confidence with the correctness of the solution, and Steele et al. (2018) highlight that confidence predicts a creative outcome. Further support comes from Topolinski and Reber (2010b) and Salvi et al. (2016) who identified a higher probability to be correct for insight solutions in convergent thinking tasks.

Happiness and, more generally, a positive mood is strongly linked to insights and Eureka experiences in the existing literature. In the “Dira” task participants experiencing a strong Eureka seldom report low happiness, but instead are consistently happier than with weaker or no Eureka experiences. The meta-review of Baas et al. (2008) provides a comprehensive overview of the relationship between mood and insight. More recently Shen et al. (2015) explore 98 different emotional states and their relationship to “Aha!” experiences. Results from their studies 2 and 3 suggest a link between insight and happiness—along with a list of other positive emotional states. The mapping of states in two dimensions affords that other emotions could mask happiness for weaker Eureka experiences. While Abdel-Khalek (2006) finds single-item measurements of happiness sufficient to assess related positive affects and emotions, the fine-grained exploration of the emotional space associated with emerging solutions could be a topic for future research.

Our results for the relationship between difficulty and Eureka show that “Dira” tasks with a strong Eureka experience are rarely perceived as difficult. This finding seems counter-intuitive from the perspective of the classical “insight sequence” (Ohlsson, 1992) in which a complicated impasse has to be navigated. However, perceived difficulty can change in hindsight. Even if the task appears to be problematic while working on it, Topolinski and Reber (2010a) have shown that having an insight can change this. In a review of the literature, they identify a change of processing fluency as a result of having an insight. After having found the solution, they conclude, the problem appears to be easier than it was during the attempt to solve it. Alternatively, yet another interpretation is that the participants experience insights in tasks that are not difficult for them.

### 5.3. Differences between conditions and personalities

In section 3.1 we provide a theoretical argument for administering “Dira” in the three different conditions. In particular, we hypothesized providing a potential solution would result in an increased interaction time. The collected data do not support this hypothesis as the results in section 4.1 show. We had further assumed that the additional task of elaborating on the chosen solution would increase the interaction time and change the self-report. As section 4.2 demonstrates, the data do not provide evidence for this effect. This could either mean that the theoretical argument is not sound and additional variables would influence the measurements to an extent that masks the hypothesized effect. Furthermore, the introduced interventions might tap into different effects than expected. Assuming that the theoretical argument is valid, the effect size could be too small or “Dira” as an instrument not sensitive enough to measure the effect within the sample. In summary, there is no evidence that supports a difference between the behavioral or self-reported measures among the three conditions.

In a trial-by-trial comparison, we reveal a link between fewer interactions and stronger Eureka experiences. In section 4.3 we compare the differences in the number of interactions observed between Eureka intensities, separately during and after the “First Full Scan.” We observe a significantly larger variance between no and strong Eureka experiences after the “First Full Scan.” This difference implies that the experience is influenced by element interactions and not by the participants' distinctive approach to the task. On the other hand, individual variability might moderate the experience and performance in the “Dira” experiment. Future research could expand the method we suggest to address the relationship with personality traits. Specifically, “Dira” could be used to test if traits known to correlate with creative production (Batey et al., 2010) predict eureka experiences.

### 5.4. Experimental control

The participants' freedom to choose the order and duration of stimulus interaction is supposed to increase task engagement, but it does not come without costs. The flexibility to look at elements in any order allows participants in the “Dira” experiment to not look at elements necessary to solve the problem. For example, some participants choose not to look at the text before selecting one of the images. Furthermore, participants who start with the text and try to find a matching image afterwards might use a different approach to solve the problem than others who engage with images first and interact with the text later during the task. In the first case, they only need to store the text itself or a derived concept in working memory to match it against each of the images they look at. In the second case instead, they need to remember up to six images and related concepts to match each of them with the text. In the current study, we filtered for rounds in which participants started with the text and removed all others. Future studies could eliminate the second case by specifying the chronology, for example by showing the text first.

As discussed earlier, the bimodal distribution of hover durations suggests that participants unblur elements for at least two different reasons. As discussed in section 3.5, participants might either intend to move the mouse pointer across by targeting elements on the other side or consciously engage with the text and images. In the current study, we assumed interactions shorter than 130 ms to represent mouse movement across elements. While these interactions were removed *post-hoc* from the current study, avoiding short unblurring could be implemented in the experimental design. The elements could only be shown clearly if the hover time exceeds the movement time predicted by Fitts' law (Soukoreff and MacKenzie, 2004).

## 6. Conclusion

In the “Dira” task, we estimate the moment of the emerging solution based on the participants' behavior and self-reports without relying on additional indicators. Like in many design and engineering problems, more than one solution is correct for this task. For “Dira” we demonstrate how behavioral data and meta-cognitive monitoring are integrated by this instrument to identify sub-processes of the creative process.

The results suggest that participants can distinguish between Eureka experiences of different strengths. Thus, our results suggest that Eureka experiences are not limited to having or not having an insight, but that the perception of this experience can have different intensity levels. Future studies should keep this in mind when assessing Eureka experiences.

Looking at the whole process of finding a solution to an ill-defined problem, people experience something early in the problem solving process that they relate to the Eureka experience. While the exact timing remains unclear, observations in “Dira” help narrowing down insight and other sub-processes. For example, before seeing all the elements in the “Dira” task, participants in our study exhibit distinctive behavior related to the strength of their reported Eureka experience. Our results suggest that immediate insights exist and can be reported by people who experience them.

The creative process is often studied indirectly through the creative product, person, or press. We propose “Dira” as an experimental platform to record behavior as Eureka experiences are happening. This instrument and future studies applying the same underlying principle can bring us another step closer to understanding the creative process.

## Ethics statement

This study was carried out in accordance with the recommendations of Plymouth University Research Ethics Policy, University Research Ethics and Integrity Committee. The protocol was approved by the Faculty Psychology Research Ethics Committee. All subjects gave written informed consent in accordance with the Declaration of Helsinki.

## Author contributions

FL: design of experiment, implementation of the experiment, data collection, data analysis and interpretation, write up; JG: design of experiment; GB: design of experiment, data analysis and interpretation, paper structure and edit.

### Conflict of interest statement

The authors declare that the research was conducted in the absence of any commercial or financial relationships that could be construed as a potential conflict of interest.

## Footnotes

^1^Dixit publisher's website http://en.libellud.com/games/dixit, last access: 2018-02-23.

^2^Wikipedia: Dixit (card game) https://en.wikipedia.org/w/index.php?title=Dixit_(card_game)&oldid=823435686, last access: 2018-04-05.

^3^http://boiteajeux.net; last access 2017-11-15.
